# HSQC spectral based similarity matching of compounds using nearest neighbours and a fast discrete genetic algorithm

**DOI:** 10.1186/1758-2946-4-25

**Published:** 2012-10-03

**Authors:** Gregory K Pierens, Steven Brossi, Zhengyi Yang, David C Reutens, Viktor Vegh

**Affiliations:** 1 Centre for Advanced Imaging, Level 2, Gehrmann Laboratories, Research Road, The University of Queensland, Brisbane, Queensland 4072, Australia

**Keywords:** HSQC, Spectral matching, Similarity, Nearest neighbours, Discrete genetic algorithm

## Abstract

HSQC spectra are routinely acquired for chemical structure analysis based on hydrogen and carbon chemical environments. Two fast HSQC peak matching algorithms have been developed; a nearest neighbour approach and a probabilistic method based on an existing discrete genetic algorithm. Both of these techniques are intended to find HSQC spectra matches that supplement information generated by established molecular fingerprint methods. Our results are compared to those calculated using a specific implementation of molecular fingerprints. The nearest neighbour and genetic algorithm-based methods ranked highly particular structures missed by molecular fingerprints. Our analysis shows that by complementing molecular fingerprint matches with our findings, a comprehensive list of matches can be identified. The refined list of compounds could be used to improve the quality of compounds used in screening libraries in the pharmaceutical industry.

## Background

Database driven chemical structure identification is common practice in drug discovery. Classification of similar compounds is based on the premise that physicochemical properties are comparable [1-3]. The mapping of specific compound properties to “fingerprints” has provided a robust method of searching large databases. Currently, database searching efficiency is constrained by the size of the database, the method used to determine similarity and the function defining match quality. To improve database searching, we have concentrated on the latter two constraints and propose a new approach of identifying similar compounds using heteronuclear single quantum coherence (HSQC) spectra.

Carbon HSQC spectra are collected routinely to confirm or elucidate molecular structure in synthetic and natural product chemistry. Experimental results are presented as 2D plots with axes defined by proton (^1^ H) and carbon (^13^C) chemical shifts. The high-intensity plot features, referred to as “peaks”, delineate directly bonded hydrogen and carbon atoms of a compound. Generally, the 2D Cartesian coordinates of the peaks are reported without any reference to intensity or peak size. The intensity of the peaks could also be included in the analysis. However, care must be taken to ensure that all data was acquired using the same acquisition parameters. Since we validate our findings using published data, in this work, peak intensities are not included as part of the spectra matching. The location of peaks provides valuable information about the chemical environment of hydrogen and carbon atoms allowing molecular structure to be inferred from the number and location of peaks which have specific distributions for each compound.

A number of metrics have been used to quantify the similarity between a compound of interest and a database of compounds allowing the best database results to be selected as possible replacements for the candidate structure. For example, compound fragments and related properties have been mapped to molecular fingerprints defined using bit strings [4]. The fingerprints capture specific information about molecular structure and specific properties of a molecule [4,5]. In bit string-based fingerprinting, the Tanimoto (T_c_) [6] and Tversky [7] coefficients have been used widely to quantify the level of similarity. Above an acceptable threshold, compounds are deemed similar and therefore have similar chemical or biological properties.

We previously outlined a method of matching HSQC spectra of small compounds motivated by evolutionary optimization [8,9]. The use of self-adaptive differential evolution allowed matching of a candidate compound HSQC peaks to individual entries of a database. However, as the number of peaks increased (i.e. larger than 20), the search space became very large, to the extent that the quality of match was not computable in a reasonable amount of time. Our new approach is aimed at increasing computational efficiency by considering three factors limiting the rate of convergence of any algorithm, the choice of the metric and method to obtain an optimal solution and the size of the search space. The outcome is a robust algorithm capable of matching spectra containing a large number of peaks rapidly on a standard desktop computer.

We improved the efficiency of our previously reported HSQC spectra matching algorithm by using a discrete genetic algorithm (DGA) implementation instead of differential evolution. We tested our new method on a compound database containing 51 HSQC spectra. The results were compared to bit string based molecular fingerprints incorporating a suitable threshold for the Tanimoto coefficient [10] and to nearest neighbour search, also known as proximity search or closest point search which is the simplest implementation of all peak matching methods.

## Results and discussion

The database of 51 HSQC spectra from our previous work [8] was used to test the efficacy of our newly developed algorithm. The actual structures of the 51 compounds are listed in Additional file 1.

### Treatment of outliers in DGA

**Figure 1:**
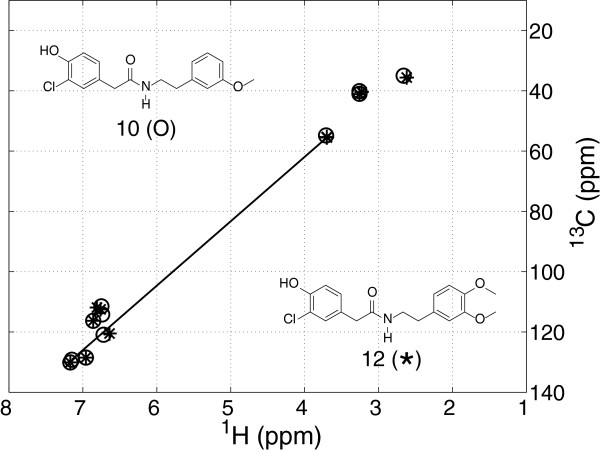
**The matched HSQC spectra for compounds 10 and 12.** The plot shows a single long distance match between two peaks leading to an amplified mean distance per peak metric value.

A problem with performing DGA-based unique matching of peaks between two spectra is that a single long match can greatly affect the outcome. An example of this problem is the peak-to-peak match of compounds **10** and **12** (Figure 1). The only difference between the structures is the number of aromatic methoxy groups; compound **12** has two and compound **10** has one with the other methoxy group being replaced by an aromatic CH. Examining the DGA matched HSQC spectra; we observed one long distance peak match and all other peaks were matched to close peaks. According to our algorithm definition, all peaks have to be uniquely matched because both compounds had 11 peaks. This resulted in an aromatic peak being matched to an aliphatic peak affecting the mean distance metric and resulting in a misclassification.

To remedy a single long distance peak-to-peak match, we identify outliers and exclude their matches in the metric used to classify match quality. We thus modified the mean distance per peak, excluding outliers by statistically examining each set of matched peak distances and applying a rejection criterion. The mean and standard deviation (σ) was calculated for all pairs of matched peaks in the HSQC spectrum to spectrum comparison. If an individual distance was greater than *S* times σ from the mean, it was considered an outlier. We then rematched any outliers to their nearest neighbour in the other spectrum. We examined several values of *S* (1.75 – 3.0) and settled on 2.5σ as the threshold value for outlier rejection. We arrived at this result by qualitatively evaluating characteristics of many spectral matches. The value of *S* is a user defined variable and can changed if unsuitable for the HSQC matching under consideration.

### Effect of population size and number of iterations in the DGA

We examined the effect of changing *K* (population size) and *G*_max_ (maximum number of generations) on convergence using the DGA method. The HSQC spectra of the 51 compounds were matched to all other spectra and the similarity metric from **p** to **q** and **q** to **p** were compared, to establish the stability of results from the algorithm. The 2601 spectral match results were recorded in a 51x51 matrix with the columns and rows corresponding to the referencing of the compounds. The upper and lower triangular parts of the matrix consisted of **p** to **q** and **q** to **p** matches, respectively. Ideally, the matrix should be symmetrical. However, since our approach is probabilistic and we limit the maximum number of iterations, corresponding entries in the upper and lower triangular sections of the matrix may differ. To examine this possibility, we compared the corresponding upper and lower triangular entries of the matrix for the three parameter sets. We considered a small (*K* = 2 and *G*_max_ = 5 *N*, where *N* is the largest of number of peaks in **p** or **q**), medium (*K* = 5 and *G*_max_ = 10 *N*) and large (*K* = 20 and *G*_max_ = 50 *N*) implementation as defined by the size of the parameters. The small parameter set was the fastest to compute with 32 differences between **p** to **q** and **q** to **p** matches, which represented an error rate of 2.5%. The medium set gave six different outcomes with an error rate of 0.5%, and the large parameter set showed only one difference with an error rate less than 0.1%. Spectra for the above data set were also matched by the SADE method and the results are shown in Table 1. Overall, DGA converged with fewer function evaluations than SADE. Taking into account convergence error and speed of the calculation, we chose the medium parameter set for the DGA matching in the rest of the analysis. Extrapolating the SADE data to an error rate of 0.5% means that ~ 10^14^ function evaluations have to be performed, in comparison to ~ 10^10^ for DGA.

**Table 1:** The number of function evaluations (NFE) and corresponding error rate for the DGA and SADE methods

**NFE**	**DGA (%)**	**SADE (%)**
1.60E + 09	2.5	5.0
7.98E + 09	0.5	2.7
1.60E + 11	0.1	2.1

In the integer optimization problem mutations and crossovers were chosen to improve performance with respect to our application, and hence, we were able to set *G*_*max*_ relatively small. The calculation of the 2601 HSQC spectral matches using the medium settings (*K* = 5 and *G*_*max*_ = 10 N) took approximately 85 minutes, which was an average of ~ 2 seconds per match which includes overheads from the GUI and reading and writing data files. The largest peak matching was between compounds **17** (17 peaks) and **18** (22 peaks), taking approximately 4 seconds. If 20,000 HSQC spectral matches were required on similar sized spectra using the medium settings, then it would take ~ 11 hours.

### Ranking of matches against molecular fingerprint (MFP)

The results of NN and DGA approaches of matching HSQC spectra were compared to those obtained using the MFP method within Open Babel: The Open Source Chemistry Toolbox [11,12]. The FP2 path-based fingerprint, which indexes small molecule fragments, was used to generate the similarity results. All compound similarities were calculated using the Tanimoto coefficient (T_c_) which ranges from 0, no similarity, to 1, maximum similarity. The selection criterion for T_c_ of equal to or greater than a value of 0.7 was used and resulted in 44 compound matches. Of these, 38 were between compounds **113**. This was not unexpected, as these compounds are part of a combinatorial library based around 2-(3-chloro-4-hydroxyphenyl) acetamide.

**Figure 2:**
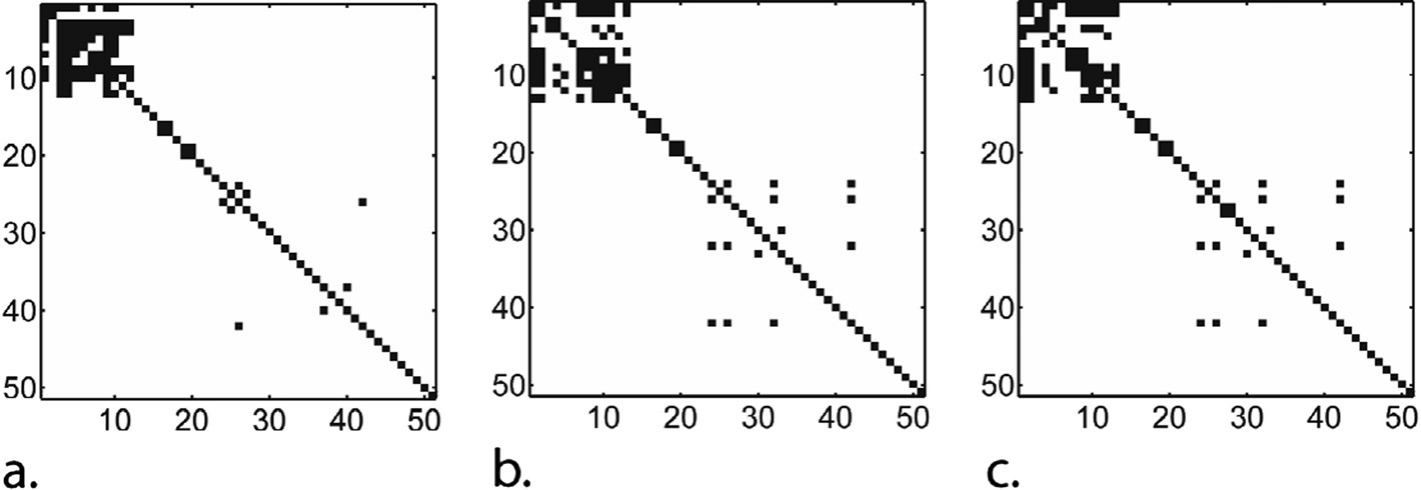
**The similarity match matrices for (a) MFP, (b) NN and (c) DGA are shown.** The off-diagonal black squares represent the top 44 ranked matches and the diagonal entries were included for reference purposes. The **p** to **q** (upper triangle) and **q to p** (lower triangle) matches are both provided.

To compare the two HSQC matching protocols (NN and DGA) with MFP, the 44 most similar HSQC spectra for each method were considered. Cut-off thresholds were 0.0228 for NN and 0.0294 for DGA (smaller number is more similar). The similarity matches for all three methods are shown in matrix form in Figure 2. As in the case of MFP, for NN and DGA, the majority of the retained matches were for compounds **1** – **13**. For compounds **20 – 45**, both NN and DGA found a larger number of matches than MFP.

We further considered all matching metrics in 6 categories. Categories and respective cut-off values are provided in Table 2. A smaller category number reflects a better match. The top three categories (1–3) were all above the threshold used for the top 44 matches and cut-offs for them were at regular intervals. The same intervals were continued below the threshold for Categories 4 and 5. Category 6 contained the rest of the matches. In the following subsections we investigate how these categories overlap amongst the various matching approaches.

**Table 2:** The categories and cut-offs for MFP, NN and DGA

**Category**	**MFP**	**NN**	**DGA**
1	[0.9, 1.0]	[0, 0.0076)	[0, 0.0098)
2	[0.8, 0.9)	[0.0076, 0.0152)	[0.0098, 0.0196)
3	[0.7, 0.8)	[0.0152, 0.0228)	[0.0196, 0.0294)
4	[0.6, 0.7)	[0.0228, 0.0304)	[0.0294, 0.0392)
5	[0.5, 0.6)	[0.0304, 0.0380)	[0.0392, 0.0490)
6	< 0.5	≥ 0.0380	≥ 0.0490

The smaller the category number, the better the expected match. The first three categories capture the 44 matches of Figure 4.

### NN versus DGA based HSQC spectra matching

Among the top 44 similarity matches, NN and DGA HSQC similarity methods identified only seven different matches. The matches unique to NN were all within compounds **1**–**13**, while for DGA, only one was from this group of compounds. All NN matches were all in category 4 for DGA, just outside the threshold to be classed as similar. Five out of the seven DGA matches were in Category 4 and two were in Category 6 from NN. The two HSQC matches for compounds **7** and **11** are provided in Figure 2. In this case the spectra classified in Category 3 for NN and Category 6 for DGA.

**Figure 3:**
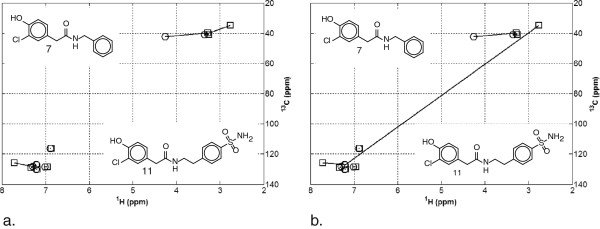
The HSQC spectra matching result of compounds 7 and 11 using (a) NN and (b) DGA.

Figure 3 illustrates the impact of the outlier rejection criterion of 2.5σ used in this DGA comparison. In this case, DGA places the match in Category 6 whereas NN places it in Category 3. If the criterion for an outlier was lowered from 2.5σ to 2.25σ, classification would change from category 6 to 3. Hence, DGA would identify them as similar HSQC spectra. The NN methodology can therefore be used to identify matches that may be overlooked in the DGA matches. We propose the use of NN and DGA in conjunction to identify and validate HSQC spectral matches.

### Comparison of MFP, NN and DGA results

**Figure 4:**
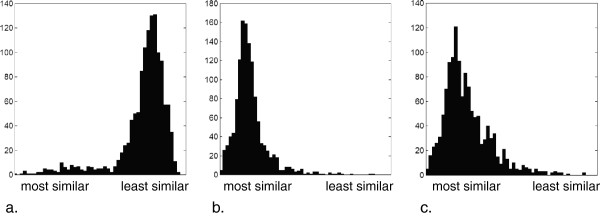
**Illustrated are histograms of the similarity measures for the 1275 comparisons.** Provided are results for (**a.**) MFP, (**b.**) NN and (**c.**) DGA.

A histogram was created from the 1275 match results of each method, as illustrated in Figure 4. There are differences in the shape of the histograms obtained using MFP (negatively skewed) as compared to the two HSQC spectral matching methods (positively skewed). For the MFP method, the region of the histogram corresponding to most similar spectra is widely spread, indicating that the method can discriminate between similar compounds. The MFP distribution shows that a large proportion of the matches are classified as dissimilar, suggesting that it is highly sensitive to changes in the bit-string fingerprint.

The NN and DGA histograms are similar with the highest frequency of scores appearing in the “most similar” region. The primary difference between MFP and the other two matching methods is that in MFP, a feature is either present or not within a fingerprint, whereas a distance between matched peaks is computed in both NN and DGA. This means that a feature is always included in NN and DGA, irrespective of whether a peak match is identified as an outlier in the latter approach. The histogram distribution is narrower for NN than for DGA. Thus is likely to be due to DGA identifying a unique peak-to-peak match, which results in an overemphasis of the peak distances. On the other hand, NN matches peaks non-uniquely, essentially providing information about the peaks’ neighbourhoods with respect to the other HSQC spectrum. NN and DGA can both suffer from false positives.

**Figure 5:**
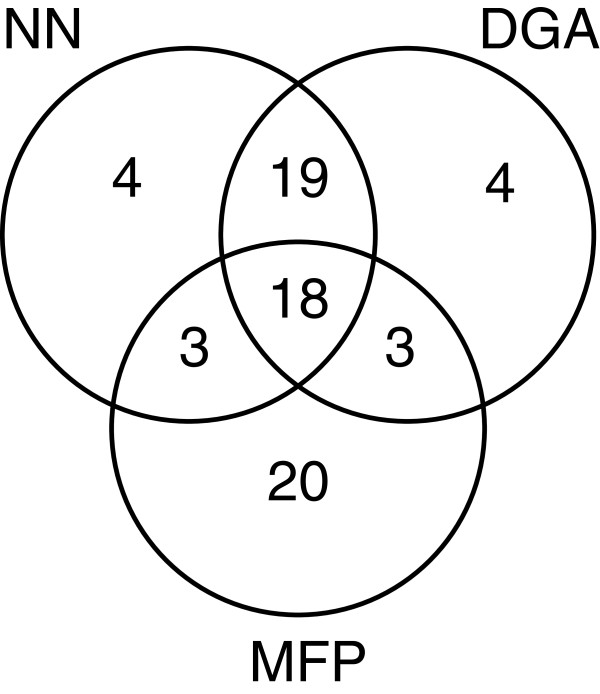
The Venn diagram showing the top 44 ranked matches and how matches relate between MFP, NN and DGA.

To simplify the interpretation of our results, the 44 most similar compounds or spectra from each methodology were compared using a Venn diagram and for the full list of similar matches refer to Additional file 1. The sets and their overlaps are provided in Figure 5.

There were 19 HSQC matches that were only common to NN and DGA. Of the 19 common matches, 14 were between spectra of compounds **1**–**13**. The other five are shown in Table 3 along with their chemical structure and ranking category. All other results are provided in the supporting information. Spectra from compounds **24** and **32** were found to be in category 1 for NN and DGA, but MFP placed it in category 4. Category 4 is just below the threshold for being classified as similar, and MFP would have disqualified it from further investigation, even though the compounds are similar from a structural point-of-view. Compound matches **24** to **42** and **26** to **32** were not identified as similar using MFP (category 5). All of these compounds have similar structural groups, but they are arranged differently around the phenyl ring. We consider these compounds to be similar based on their structures.

**Table 3:** Illustrated are specific compound structures found to be similar using NN and DGA and not using MFP

**Compound A**	**Compound B**	**Categories**		
		**MFP**	**NN**	**DGA**
24	32	4	1	1
24	42	5	2	2
26	32	5	2	2
30	33	4	3	3
32	42	4	2	3

In view of our findings, we recommend the following protocol for matching of HSQC spectra. First, calculate MFP, NN and DGA based similarities. Determine the MFP cut-off to be used; this is usually set to 0.7. Calculate the number of structures identified by the MFP method and set a suitable threshold to obtain the same number of structures using NN and DGA in accordance with their ranking. The highly significant compound structures would be matches identified by at least two of the methods. In our case, this would be 43 [18(common to all), 3(MFP and NN only), 3(MFP and DGA only) and 19(NN and DGA)]. The compounds that were identified only by one method should be reviewed on a case-by-case basis.

## Conclusions

The research aimed to investigate whether new approaches can improve a molecular fingerprint-based method of identifying structurally similar compounds from databases of HSQC spectra. Two fast peak-to-peak spectral matching methods were developed, the nearest neighbour and discrete genetic algorithm methods. We found that complementary information from both methods improved the classification of compound structures. We compared our new approaches to a method based on molecular fingerprints, and investigated differences between matches. We conclude that our approaches are not a replacement for existing established methods; instead they should be used to refine the assessment of similarity. The use of our algorithms can help counter missed similarity matches arising when molecular fingerprint is used solely for matching of HSQC spectra.

## Methods

The *n*^th^ peak in a 2D HSQC spectrum is defined as a feature point, denoted as:

(1) $$p_{n}=\left(x_{n},y_{n}\right)\text{,}$$

where *x*_*n*_ and *y*_*n*_ are the real valued Cartesian coordinates for the normalized ^13^C and ^1^ H chemical shifts, respectively. A spectrum is then defined as a set of points:

(2) $$p=\left\{p_{n}:n=1,2,\ldots ,N\right\}\text{,}$$

where *p*_*n*_ is the coordinate of the *n*^th^ peak and *N* is the number of peaks. Given another spectrum $q=\left\{q_{m}:m=1,2,\ldots ,M\right\}\text{,}$ the goal is then to match peaks by minimizing a metric quantifying the quality of match between **p** and **q**.

We first define a positive metric for two peaks *p*_*n*_ and *q*_*m*_:

(3) $$d_{n,m}=\left\|p_{n}-q_{m}\right\|\text{,}$$

and the error in the match when all peaks are considered is:

(4) $${ɛ}_{s}\left(j\right)=\sum_{n=1}^{N}{\hat{d}}_{n,j_{n}},{\hat{d}}_{n,j_{n}}=\{\begin{array}{c} d_{n,j_{n}}\\ 0 \end{array}\begin{array}{c} j_{n}<M\\ \text{otherwise} \end{array}\text{,}$$

where **j** is a vector of *N* elements and $j_{n}\in \left[1,M\right]$ is a perturbation on *m* given *n*, such that ε is minimized when **j** is the optimal indexing of **q**. The term $\varepsilon_{S}$ measures the quality of match when all peaks are matched. In the case when one spectrum contains more or less peaks than the other, all peaks from the smaller spectrum are matched, leaving some peaks in the larger spectrum unmatched. We will use the “matched” and “unmatched” terminology throughout this paper. If *N* < *M*, **j** contains *N* unique integers in [1,*M*], and hence, the unmatched peaks of **q** do not appear in **j**. If *N* > *M*, then **j** contains *N* unique integers from [1,*N*]. As such, the entries where *j*_n_ > *M* are left unmatched. The modified metric, $\hat{d}$, accounts for this case.

### Nearest Neighbour (NN) matching

**Figure 6:**
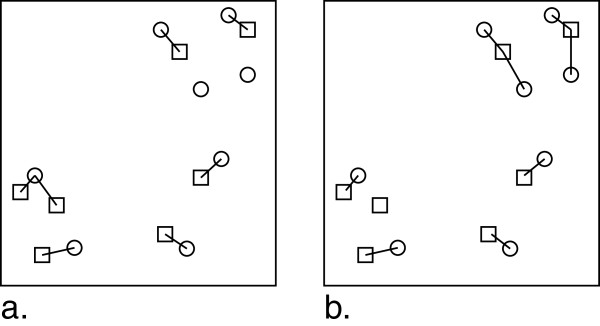
**The schematic outlines the matching process of HSQC spectra using the nearest neighbour method.** Illustrated are (**a**) nearest neighbour matchings from squares to circles and (**b**) nearest neighbour matchings from circles to squares. The average distance per peak is calculated by averaging all matched distances.

A nearest neighbour HSQC similarity match was computed where each peak of **p** is matched to the nearest peak of **q** and each peak of **q** was matched to the nearest peak in **p**. Furthermore, an average distance-per-peak metric was used, as illustrated in Figure 6. The NN based matching can result in a single peak being matched to many peaks from the other spectrum. Therefore, it gives an indication of relative clustering of peaks. Overall, NN based matching of HSQC spectra is computationally efficient and provides a deterministic result.

The NN approach does not take into account different numbers of peaks in different areas of the spectrum. This can result errors in ascribing compound similarity on the basis of HSQC spectra. For this reason, we also propose to uniquely match spectra peaks, enabling improved differentiation of compound structures through the introduction of long distance peak matching in the metric. This type of matching implemented in our previous work using differential evolution had the drawback that establishing matches to database entries with more than 20 HSQC spectra peaks was time consuming. Our improved method based on a discrete genetic algorithm is still probabilistic and obtains good approximations for large numbers of peaks in a practical amount of time.

### Discrete genetic algorithm (DGA) matching

We use a discrete genetic algorithm to optimize the optimal indexing in (4). Our implementation was inspired by the algorithm applied to solve traveling salesman problems. In this work we closely followed the implementation outlined by Schneider [13]. We defined *K* to be the population size (i.e. the number of solutions) and *G*_max_ as the maximum number of generations.

Our DGA implementation did not involve forcing of match directions. That is, given a spectrum **p** to be matched to **q**, we did not require the denotation of spectrum to be such that **q** always had a larger number of peaks than **p**. Furthermore, we used injection of sort solutions through progressive iterations of the algorithm, and when the number of peaks in **p** and **q** were differed, we left |*N – M*| peaks unmatched. The following mutations were used in DGA:

• EXC(L2O): Select two peak matches and exchange them;

• L3O: Select three peak matches and shuffle them such that none of them are the same as the starting point;

• L4O: Same as L3O but using four peak matches;

• EXON: Used only when *N* < *M*. Exchanges a peak match of set **s** with a number from [1,*M*] that is not an element of **s**, hence named *EX*change with *O*utside *N*ode.

We updated the population using five mutation sweeps using: RX, BURTRAND and SINGLEBURST crossovers [13]:

• RX (random): **r** is a string of independent random bits of length *N*, with equal probabilities for zero and one.

• BURSTRAND: Same as above but with dependence between the bits such that P(**r**(*i* + 1) ~ = **r**(*i*)) = 2/*N*, where P denotes probability. This way of generating “perturbation” or noise is often used for simulating bursty channels (also known as Gilbert–Elliott channel).

• SINGLEBURST: **r** is a continuous block of ones. The length is chosen randomly in [3, *N*] and the start position *i* is chosen randomly in [1, *N*]. The block rolls over when *i* + l > *N*, such that **r**(1 to (l + *i*-*N*)) = 1.

**Figure 7:**
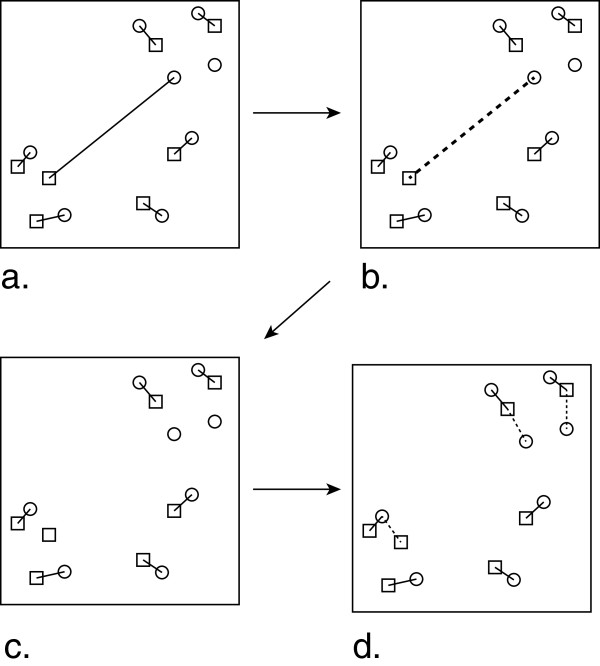
**The schematic outlines the HSQC spectra matching process using the DGA.** Illustrated are (**a**) unique peak-to-peak matching (solid lines), (**b**) identification of outliers (bold dotted line), (**c**) removal of outlier match and (**d**) matching of unmatched peaks to nearest neighbours from the other spectrum (dotted lines).

DGA minimizes (4), the sum of all peak-to-peak distances constituting a matching. For comparing the similarity of compounds we extend this concept further by introducing three levels of the metric. The first level is a unique match between two spectra, where |*N – M*| unmatched peaks are not penalized. The second level involves the identification of outliers, as determined from a single individual large distance, and the removal of these connections. The third level is the application of a penalty to unmatched peaks. This process is outlined in Figure 7.

We provide the functions of DGA, description of terms and detailed explanation of our specific metric implementation can be found in Additional file 1.

## Competing interests

The authors declare that they have no competing interests.

## Authors’ contributions

GP: Conceived idea, developed methodology and wrote paper SB: Developed methodology and Matlab programming ZY: Developed methodology DR: Developed methodology VV: Developed idea, methodology and wrote paper. All authors read and approved the final manuscript.

## Supplementary Material

Additional file 1 Contains the structures of the 51 compounds in the database, detailed information on the Discrete Genetic Algorithm (DGA) Algorithm and the comparison of Similarity classification for top44 matches in MFP, NN and DGA. Click here for file

## References

[B1] Johnson MA Maggiora GM Concepts and applications of molecular similarity 1990 John Wiley & Sons, New York

[B2] Martin YC Kofron JL Traphagen LM Do structurally similar molecules have similar biological activity J Med Chem 2002 45 4350 4358 10.1021/jm020155c 12213076

[B3] Patterson DE Cramer RD Ferguson AM Clark RD Weinberger LE Neighborhood behavior: a useful concept for validation of "molecular diversity" descriptors J Med Chem 1996 39 3049 3059 10.1021/jm960290n 8759626

[B4] Eckert H Bajorath J Molecular similarity analysis in virtual screening: foundations, limitations and novel approaches Drug Discov Today 2007 12 225 233 10.1016/j.drudis.2007.01.011 17331887

[B5] Willett P Similarity-based virtual screening using 2D fingerprints Drug Discov Today 2006 11 1046 1053 10.1016/j.drudis.2006.10.005 17129822

[B6] Willett P Barnard JM Downs GM Chemical similarity searching J Chem Inf Comput Sci 1998 38 983 996 10.1021/ci9800211

[B7] Tversky A Features of similarity Psychol Rev 1977 84 327 352

[B8] Pierens G Mobli M Vegh V Effective protocol for database similarity searching of heteronuclear single quantum coherence spectra Anal Chem 2009 81 9329 9335 10.1021/ac901616t 19856946

[B9] Vegh V Pierens GK Tieng QM A variant of differential evolution for discrete optimization problems requiring mutually distinct parameters International Journal of Innovative Computing, Information and Control 2011 7 897 914

[B10] Bajorath J Integration of virtual and high-throughput screening Nat Rev Drug Discovery 2002 1 882 894 10.1038/nrd941 12415248

[B11] Guha R Howard MT Hutchison GR Murray-Rust P Rzepa H Steinbeck C Wegner J Willighagen EL The blue obelisk interoperability in chemical informatics J Chem Inf Model 2006 46 991 998 10.1021/ci050400b 16711717 PMC4878861

[B12] The open babel package, version 2.3.0 http://openbabel.sourceforge.net/

[B13] Schneider JJ Kirkpatrick S Application of genetic algorithms to TSP Stochastic optimization 2006 Springer, Berlin Heidelberg 415 422 Scientific Computation

