# Supplementary material for: HSQC spectral based similarity matching of compounds using nearest neighbours and a fast discrete genetic algorithm
Source: J Cheminform. 2012 Oct 3;4:25. doi: 10.1186/1758-2946-4-25 (PMC3582538; doi:10.1186/1758-2946-4-25)
Supplement: Additional file 1 — Contains the structures of the 51 compounds in the database, detailed information on the Discrete Genetic Algorithm (DGA) Algorithm and the comparison of Similarity classification for top44 matches in MFP, NN and DGA. [file 1758-2946-4-25-S1.pdf]

Additional file 1

HSQC Spectral Based Similarity Matching of Compounds using Nearest Neighbours and a Fast Discrete Genetic Algorithm

Gregory K. Pierens*, Steven Brossi, Zhengyi Yang, David C. Reutens and Viktor Vegh

Centre for Advanced Imaging, University of Queensland, Brisbane, Queensland 4072, Australia

* Corresponding author:

Gregory K. Pierens, Centre for Advanced Imaging, Level 2, Gehrmann Laboratories, Research Road, The University of Queensland, Brisbane, Queensland, Australia 4072. Email: greg.pierens@cai.uq.edu.au

Abstract

HSQC spectra are routinely acquired for chemical structure analysis based on hydrogen and carbon chemical environments. Two fast HSQC peak matching algorithms have been developed; a nearest neighbour approach and a probabilistic method based on an existing discrete genetic algorithm. Both of these techniques are intended to find HSQC spectra matches that supplement information generated by established molecular fingerprint methods. Our results are compared to those calculated using a specific implementation of molecular fingerprints. The nearest neighbour and genetic algorithm-based methods ranked highly particular structures missed by molecular fingerprints. Our analysis shows that by complementing molecular fingerprint matches with our findings, a comprehensive list of matches can be identified. The refined list of compounds could be used to improve the quality of compounds used in screening libraries in the pharmaceutical industry.

Table of Contents:

S2. Structures of the 51 compounds in the database
S3. Discrete Genetic Algorithm (DGA) Algorithm
S4. Comparison of Similarity classification for top44 matches in MFP, NN and DGA

S2. Figure S-2. Structures of the 51 compounds in the database

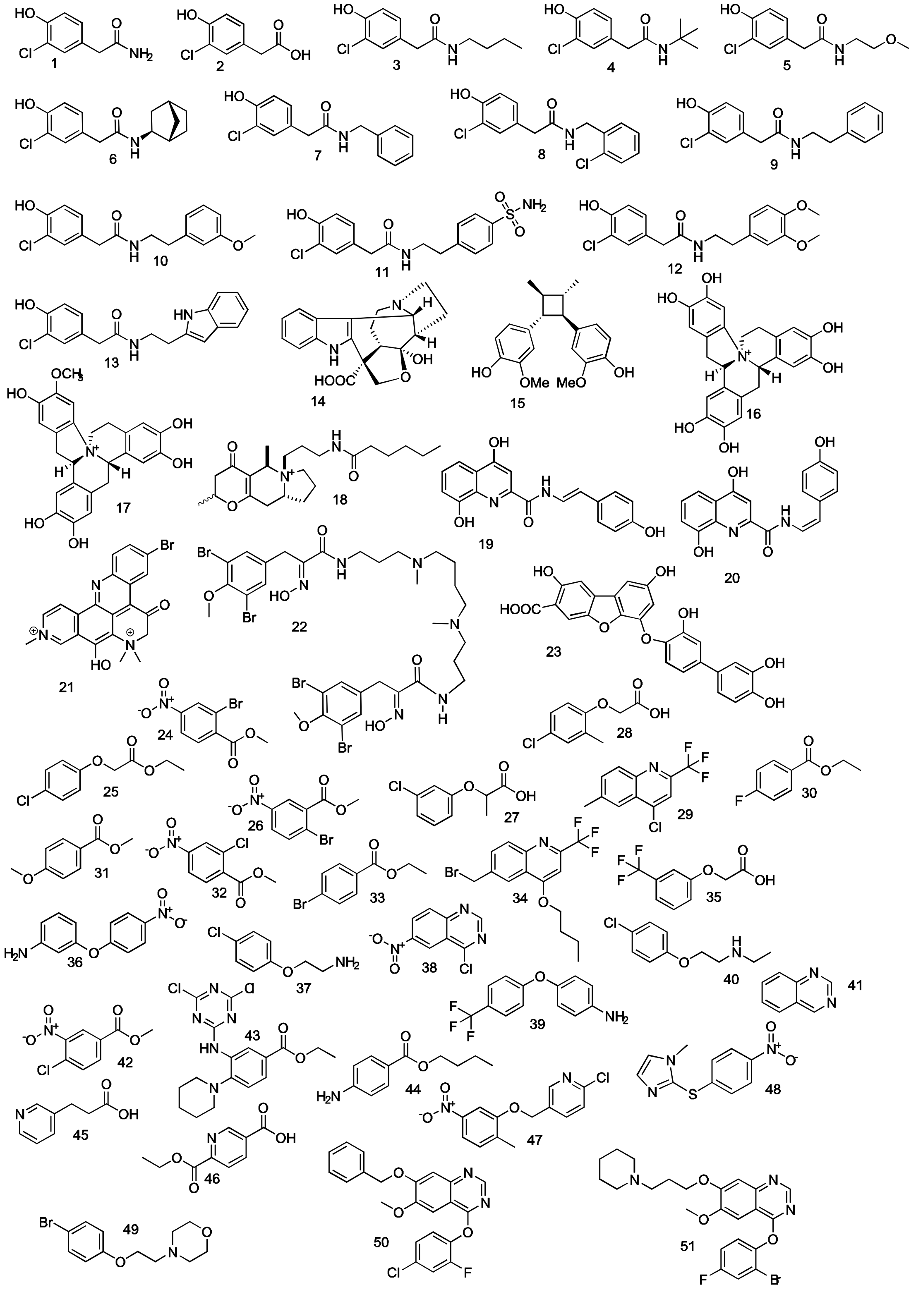

S3: Discrete Genetic Algorithm (DGA) Algorithm

We modified the discrete genetic algorithm implementation of Schneider *et al.** to solve HSQC spectra matching problems. First we provide the key features of our implementation followed by a detailed description of the specific components of the HSQC spectra matching algorithm. We used MATLAB® syntax to define individual functions.

S3.1 Algorithm features

Our implementation utilizes:

1. No forcing of match direction: we do not swap P and Q such that the smaller number of peaks is matched to the larger number of peaks.
2. Injection of sort solutions: we use a sorted solution as part of the population.
3. Unmatched peaks: leave the difference in number of peaks unmatched when the number of peaks in Q is greater than in P.
4. Mutations: we used all of Schneider's mutations (EXC, NIM/SHIFT, L3O, L4O, EXON).
5. Refining of population: five mutation sweeps.
6. Crossovers: random, burstrand and singleburst.
7. Three levels of similarity measurement: uuAMD, cuAMD, and ctAMD.

S3.2 Main function

```
FUNCTION dga_sm         {discrete genetic algorithm - similarity matching}
input: p, q (global)
s(1:N,1:K) = initialize_solutions() {sort solutions plus random ones}

loop g = 1:Gmax*N
      loop k = 1:K            {polish solutions}
            loop i = 1:5      {five mutation sweeps}
                  s_tilde = mutate( s(1:N,k) )
                  if eps(s_tilde) < eps( s(1:N,k) )
                        s(1:N,k) = eps(s_tilde)
                  end
            end
      end
      loop k = 1:K            {gender}
            mum = s(1:N,k)
            dad = select_father(s, k)
            c(1:N, k) = cross(mum, dad)
      end
      s = merge_kill(s, c)    {remove half of the solutions to retain
original population size}
end
k_hat = argmin_k (eps(s(1:N, k))
j = s(1:N, k_hat)
```

eps is defined in eqn 4 in the manuscript

*Schneider, J. J.; Kirkpatrick, S. In Stochastic Optimization; Springer Berlin Heidelberg, 2006, p 415-422

S3.3 Functions called by dga_sm

`FUNCTION initialize_solutions`

`input: {uses only global` **`p, q`**`}`

If N = M, then create one solution by sorting **p** and **q** along their ^{1}H coordinates and matching the peaks according to their order plus another solution using the ^{13}C coordinates in the same manner. If N ~= M, then two solutions are created from each coordinate, one by starting to match at the beginning of the two sorted lists and one by starting at the end. If N > M, the N - M remaining entries are set to N+1, N+2, ..., M. {Note: $\mathbf{j}_n$ > M means that p_n is unmatched.}

The remaining solutions are initialized by randomly shuffling the sequence of integers from 1 to N.

`FUNCTION mutate`

`input: s`

This function changes the solution (s) by one of the following mutations with equal probability, where function randi(a,b) draws a uniformly distributed random integer number from [a,b].

```
NIM (SHIFT)   {Shifts a portion of s by removing and re-inserting a position}
      a = randi(1, N)
      b = randi(1, N)   {a=b is not allowed, repeated in real code}
      temp = s_a
      if  a<b
            s_a:b-1 = s_a+1:b
      end
      if a>b
            s_b+1:a = s_b:a-1
      end
      s_b = temp
```

L2O (EXC): Selects two positions and exchanges them.
L3O: Selects three positions and shuffled them such that none ends up at the same place.
L4O: Selects four positions and shuffled them such that none ends up at the same place.
EXON: Only if N<M. Exchanges a position of s with a number from [1,M] that is not an element of s. Hence, it is named EXchange with Outside Node.

`FUNCTION select_father`

`input:` **`s`**`, k`

Ranks the remaining solutions (i.e. all except k) and picks solution with rank r = ceil(rand^2 * K), where rand is a random number drawn from a uniform distribution over (0,1), and ceil rounds up to an integer. This gives priority to better solutions.

`FUNCTION cross`

`input:` *`mum`*`,` *`dad`*

We use three different crossover schemes with equal probability. They all clear a certain portion of the positions of *mum* defined by the '1's of a bit string **r**. Then these positions are filled with entries from *dad* that are not in *mum* (after clearing), processed in the order in which they appear in *dad*. This implementation is the same as that given by Schneider *et al*, with the exception that if N < M, a crossover may introduce nodes that were not in *mum* prior to the application of the crossover.

Crossover schemes:

1. RX (random): **r** is a string of independent random bits of length N, with equal probabilities for '0' and '1'.
2. BURSTRAND: Same as above, but with dependence between the bits such that P(**r**(i+1)~=**r**(i)) = 2/N, where P(.) denotes probability. This approach of generating a “perturbation” or noise is often used for simulating bursty channels, also known as the Gilbert–Elliott channel.
3. SINGLEBURST: In this case **r** has a continuous block of '1's. The length of l’s is chosen randomly in [3, N] and the start position (i) is chosen randomly in [1, N]. The block rolls over in the case of i+l > N, hence **r(**1:(l+i-N)) = 1.

```
FUNCTION merge_kill
input: s, c
```

Combines **s** (parent solutions) and **c** (child solutions) and removes half of the new population with the largest eps.

S3.4 Similarity measurements

The dga_sm algorithm minimizes eps, that is the sum of all peak-to-peak distances constituting to the overall matching. To compare HSQC spectra, we extend this concept further by introducing three levels of similarity measurements:

1. Uncorrected unique average matching distance (uuAMD): The average of the distances from uniquely matched peaks as given by **j**:

```
uuAMD =     eps(j)/N,          if N<=M
            eps(j)/M,          otherwise
```

2. Corrected unique average matching distance (cuAMD): As above, but we ignore distances identified to be outliers. That is, remove distance larger than mean + X times the standard deviation. We create **j_tilde** by setting the entries of **j** that result in matches, which exceed this limit, to M+1 (i.e. unmatched). Then:

```
cuAMD =     eps(j_tilde)/N,          if N<=M
            eps(j_tilde)/M,          otherwise
```

3. Corrected total average matching distance (ctAMD): As above, but include a penalty for all peaks that are unmatched (whether due to unequal number of peaks between the two spectra or rejection of outliers). The penalty for each unmatched peak is the distance to its nearest neighbour in the other spectrum. We take the average over the total number of peaks, thus we can write the ctAMD as:

$$\text{ctAMD} = (2*\text{eps}(\mathbf{j\_tilde}) + \text{sum}(u \text{ element-of } u_p)(d_{u,NNq(u)}) + \text{sum}(u \text{ element-of } u_q)(d_{NNp(u),u})) / (M+N)$$

where u_p is the set of unmatched peaks in **p**, u_q is that of unmatched peaks in **q**, $NN_q(u)$ denotes the nearest neighbour of u in **q** and $NN_p(u)$ that in **p**.

S4. Comparison of Similarity classification for top44 matches in MFP, NN and DGA

Common to methods

Compound A	Compound B	MFP rank	NN rank	DGA rank
**1**	**2**	3	1	1
**1**	**4**	2	3	3
**1**	**7**	3	2	2
**1**	**9**	2	2	1
**1**	**10**	3	3	2
**3**	**4**	1	3	2
**4**	**9**	1	3	3
**4**	**11**	3	3	3
**7**	**8**	1	1	1
**7**	**9**	3	2	2
**8**	**9**	3	2	2
**9**	**10**	2	2	3
**9**	**11**	3	1	1
**10**	**12**	1	1	1
**16**	**17**	1	2	1
**19**	**20**	1	2	2
**24**	**26**	3	3	2
**26**	**42**	3	2	2

Common only to MFP and NN

Compound A	Compound B	MFP rank	NN rank	DGA rank
**5**	**10**	3	3	4
**7**	**10**	3	3	4
**9**	**12**	2	3	4

Common only to MFP and DGA

Compound A	Compound B	MFP rank	NN rank	DGA rank
**1**	**5**	3	4	3
**4**	**6**	2	4	3
**4**	**10**	2	4	3

Common only to NN and DGA

Compound A	Compound B	MFP rank	NN rank	DGA rank
**1**	**8**	4	2	2
**1**	**11**	4	2	2
**1**	**13**	5	3	3
**2**	**7**	5	2	2
**2**	**8**	5	2	2
**2**	**9**	4	2	2
**2**	**10**	5	3	3
**2**	**11**	6	2	2
**2**	**13**	6	3	3
**5**	**12**	4	3	3
**9**	**13**	4	3	3
**10**	**11**	4	2	2
**10**	**13**	4	3	3
**11**	**13**	5	3	3
**24**	**32**	4	1	1
**24**	**42**	5	2	2
**26**	**32**	5	2	2
**30**	**33**	4	3	3
**32**	**42**	4	2	3

Unique to MFP

Compound A	Compound B	MFP rank	NN rank	DGA rank
**1**	**3**	3	6	6
**3**	**5**	3	6	6
**3**	**6**	2	4	4
**3**	**7**	3	6	6
**3**	**8**	3	6	6
**3**	**9**	1	5	6
**3**	**10**	2	5	6
**3**	**11**	3	5	6
**3**	**12**	3	6	6
**4**	**5**	2	6	6
**4**	**7**	2	4	5
**4**	**8**	3	4	5
**4**	**12**	3	4	4
**5**	**6**	3	6	6
**5**	**7**	3	4	6
**5**	**9**	2	4	6
**6**	**9**	2	6	6
**6**	**10**	3	6	6
**25**	**27**	3	4	6
**37**	**40**	2	5	4

Unique to NN

Compound A	Compound B	MFP rank	NN rank	DGA rank
**7**	**11**	4	3	6
**7**	**13**	5	3	4
**8**	**11**	4	3	6
**11**	**12**	4	3	4

Unique to DGA

Compound A	Compound B	MFP rank	NN rank	DGA rank
**1**	**12**	4	4	3
**2**	**4**	4	4	3
**2**	**12**	5	4	3
**27**	**28**	4	4	3
